# Detection of Pilots’ Psychological Workload during Turning Phases Using EEG Characteristics

**DOI:** 10.3390/s24165176

**Published:** 2024-08-10

**Authors:** Li Ji, Leiye Yi, Haiwei Li, Wenjie Han, Ningning Zhang

**Affiliations:** 1 School of Mechatronics Engineering, Shenyang Aerospace University, Shenyang 110136, China; 2 Key Laboratory of Rapid Development & Manufacturing Technology for Aircraft, Shenyang Aerospace University, Ministry of Education, Shenyang 110136, China; 3 Shenyang Aircraft Corporation, Shenyang 110136, China; lihaiwei007@126.com (H.L.); hwj623@126.com (W.H.); 4 School of Design & Art, Shenyang Aerospace University, Shenyang 110136, China; znninging@aliyun.com

**Keywords:** beta wave, EEG map, sample entropy, Shannon entropy, psychological workload, SVM

## Abstract

Pilot behavior is crucial for aviation safety. This study aims to investigate the EEG characteristics of pilots, refine training assessment methodologies, and bolster flight safety measures. The collected EEG signals underwent initial preprocessing. The EEG characteristic analysis was performed during left and right turns, involving the calculation of the energy ratio of beta waves and Shannon entropy. The psychological workload of pilots during different flight phases was quantified as well. Based on the EEG characteristics, the pilots’ psychological workload was classified through the use of a support vector machine (SVM). The study results showed significant changes in the energy ratio of beta waves and Shannon entropy during left and right turns compared to the cruising phase. Additionally, the pilots’ psychological workload was found to have increased during these turning phases. Using support vector machines to detect the pilots’ psychological workload, the classification accuracy for the training set was 98.92%, while for the test set, it was 93.67%. This research holds significant importance in understanding pilots’ psychological workload.

## 1. Introduction

The “Statistical Bulletin on the Development of the Civil Aviation Industry in 2021” highlights the rapid growth in aircraft utilization across multiple sectors. In 2021, the civil aviation industry achieved notable metrics, including a total transport turnover of 59.928 billion tonne-kilometers, a passenger turnover of 391.387 billion person-kilometers, and 6.28 million completed flight hours for transport airlines. As the number of flights increases, ensuring safety remains a paramount concern in the contemporary airline industry [1].

**Figure 1:**
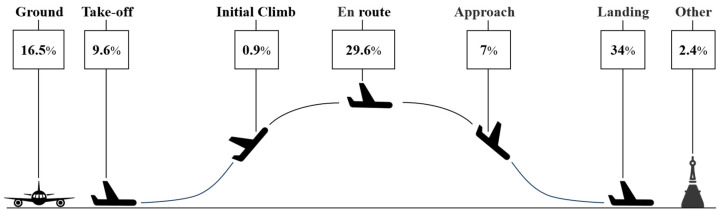
Percentage of fatal accidents.

Safety is of paramount importance in the contemporary airline industry, especially with the increasing number of flights. According to statistics from the Aviation Safety Network, Figure 1 illustrates the likelihood of accidents at different stages of an airplane’s navigation process. Although the cruise phase has a comparatively lower accident probability—around 30 percent compared to other phases—aviation safety hazards during this phase can jeopardize the lives and property of passengers, leading to unpredictable consequences [2].

**Figure 2:**
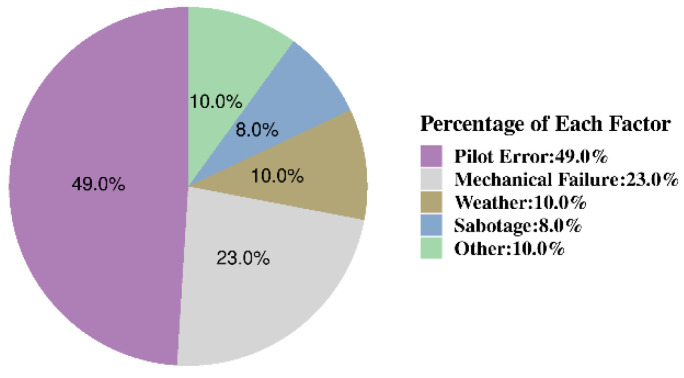
The proportion of factors causing accidents.

Among the numerous factors affecting aviation safety, pilot behavior stands out as one of the most significant. As shown in Figure 2, pilots must maintain prolonged concentration during flight missions [3]. Therefore, studying pilots’ behavior and identifying underlying patterns has become an urgent priority to enhance aviation safety and reduce the occurrence of accidents.

Enhancing pilots’ flight skills stands out as the pivotal measure to reduce the probability of flight accidents and consequently improve flight safety [4]. Continuous monitoring of pilots’ operational processes during flight training is imperative to ensure the timely correction of any irregular operations. Standardizing pilots’ operations is essential to mitigate the safety risks associated with pilot error [5].

Traditionally, flight performance assessment has relied solely on the instructor’s subjective experience. This method’s drawback lies in its susceptibility to the instructor’s subjective influence at any given moment, potentially leading to inconsistent assessment standards. To address this, scholars have proposed various assessment methods based on traditional flight training systems that utilize monitoring data to refine pilot training techniques [6,7].

Recently, researchers began assessing pilots’ conditions during flight training using physiological signal data. Li Jie et al. examined the impact of hypoxic training during high-altitude flights on pilots’ electrocardiographic (ECG) activity and concluded that ECG could effectively monitor the pilots’ training status [8]. Additionally, M. Dilli Babu independently gauged the cognitive load of pilots based on ocular parameters in a military aviation context, finding a significant correlation between pilots’ cognitive load and eye parameters [9].

Electroencephalography (EEG) is a technique for recording electrical activity generated by neurons in the brain, detected on the surface of the scalp. Compared to ECG and EOG, EEG signals offer more direct insights into the brain’s activity changes [10]. EEG has been extensively utilized in various studies to explore different aspects of cognitive and psychological states. G. F. Wilson’s research revealed that while both ECG and EEG data can indicate the pilots’ psychological workload, EEG is more reliable as it directly records the brain’s evoked potentials, providing a clearer reflection of brain activity [11]. Bartosz Binias et al. investigated the correlation between brainwave activity and reaction time in short-haul pilots using EEG data [12,13]. Additionally, Peng Zhang et al. examined brain attributes related to pilots’ error awareness during flight missions, employing EEG in their study [14]. Zhendong Lan et al. analyzed EEG characteristics during fatigue driving and classified fatigue states using support vector machines (SVM) [15]. These studies collectively underscore the importance of EEG in understanding cognitive and psychological states, offering valuable contributions to the field of pilot performance and safety.

This study aims to process and analyze EEG data collected from pilots during left and right turns in simulated flight scenarios. The analysis includes examining the changes in EEG signals, variations in EEG power, and the correlations between EEG power and turning maneuvers. Additionally, NASA-TLX is used to assess the pilots’ psychological workload during cruising and turning phases, and support vector machine (SVM) algorithms are employed to classify their psychological workload. The expected outcome is that applying neuroscience research to flight performance will provide a theoretical foundation for flight training methods, ultimately enhancing flight safety.

## 2. Materials and Methods

### 2.1. Participants

The study involved ten professionally trained male pilots, each with at least 50 h of flight simulation training. These participants met the following specific criteria to ensure the consistency and reliability of the experiment:

1. No prior EEG experiment participation: participants had no history of participating in EEG experiments, ensuring that their brain activity recordings were not influenced by prior experience.
2. Good physical health: all pilots were in good physical health, crucial for accurate EEG readings and experimental reliability.
3. Age: participants were aged between 20 and 24 years old, with an average age of 22 years (±2).
4. Right-handedness: right-handedness was a requirement, possibly to maintain consistency in motor responses or due to established correlations between handedness and brain activity.
5. Socio-economic status: participants were from a middle-class socio-economic background.

In order to optimize conditions and minimize external factors [16], the following conditions were observed:

1. Timing: the experiment was scheduled for 9:00 a.m. to minimize the impact of drowsiness, ensuring participants were alert and well-rested.
2. Weather and environmental conditions: the weather was clear, with no wind or other natural factors that could affect the flight simulation or participants’ performance.
3. Health precautions: pilots were instructed to maintain proper eating habits, get adequate sleep, and avoid alcohol or medication for three days before the experiment.

Before the experiment began, participants received a comprehensive briefing on the flight process and experimental equipment, including the EEG cap and other devices. Ethical considerations were also addressed: all participants voluntarily agreed to take part in the experiment and signed a “Consent to Participate in the Experiment” form. At the conclusion of the study, participants were compensated according to the terms outlined in the consent form, ensuring transparency and fairness in their involvement.

These measures collectively aimed to create the optimal conditions for studying EEG data during flight simulation, ensuring that the results would be reliable and applicable to enhancing aviation safety through a better understanding of pilot behavior and psychological workload.

### 2.2. Experimental Equipment

**Figure 3:**
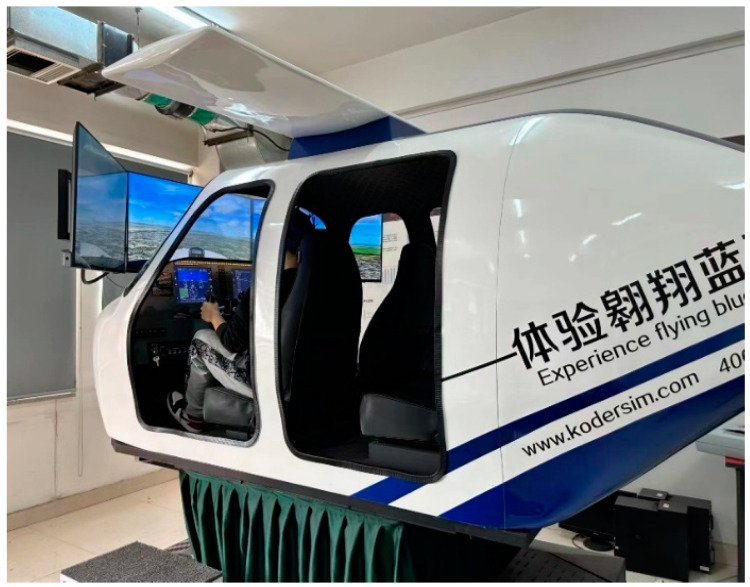
Professional flight simulator.

**Flight Simulator:** The study utilized a KDKJ-FX-172-6D four-seat Cessna professional flight simul ator. The flight simulator features a six-degree-of-freedom kinetic platform, an accurate aerodynamic model, real-time flight sound effects, a full-scale aircraft joystick, and a variety of virtual terrains to ensure that the flight simulation process is as similar as possible to the actual flight process (as depicted in Figure 3).

**EEG cap:** In this study, the Emotiv EPOC+ EEG cap was used to record EEG data during pilot driving scenarios. The device comprises fourteen data acquisition channels (AF3, F3, F7, FC5, T7, P7, O1, AF4, F4, FC6, F8, T8, P8, O2), as shown in Table 1.

**Figure 4:**
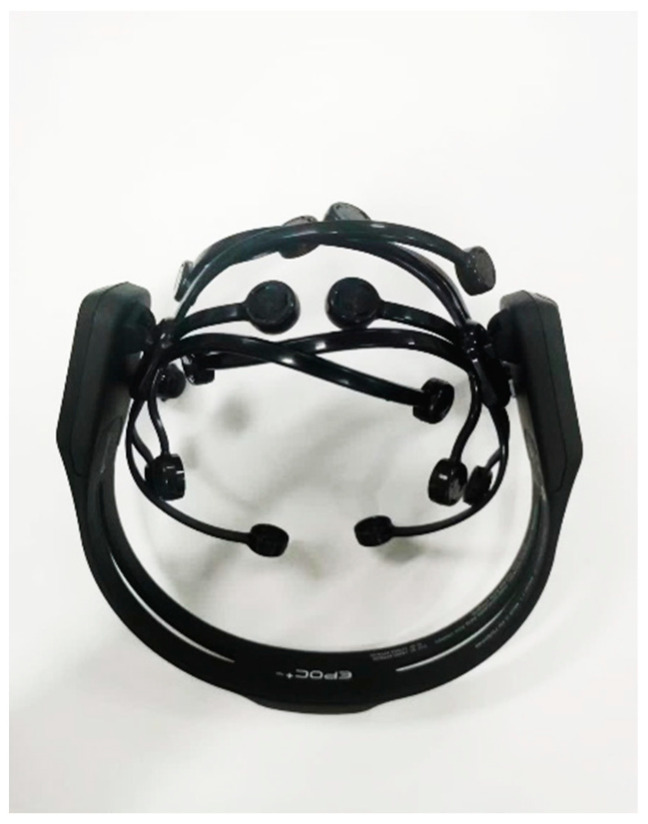
Emotiv EPOC+ EEG cap.

This EEG cap supports wireless transmission, commonly used for recording action EEG and mitigating the impact of EEG caps on pilots (as depicted in Figure 4) [17]. The cap’s ability to transmit data wirelessly reduces movement restrictions on pilots, making it ideal for dynamic and realistic simulation environments.

**Psychological Workload Questionnaire:** The NASA-TLX (Task Load Index) is a cognitive load assessment scale developed by NASA. It is a widely recognized subjective psychological workload assessment tool. The scale encompasses six dimensions: mental demand, physical demand, temporal demand, performance, effort, and frustration.

### 2.3. Experimental Steps

**Figure 5:**
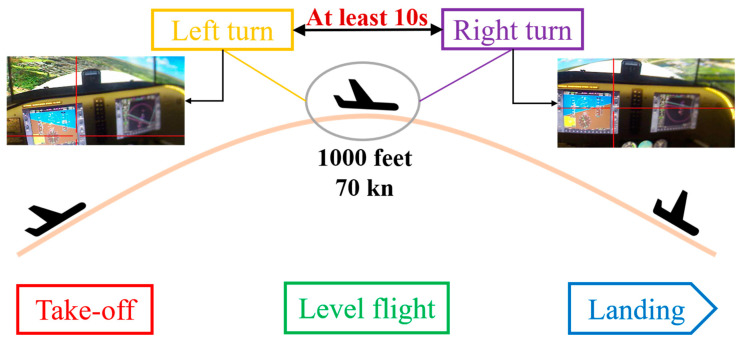
Flight simulation experiment.

The KDKJ-FX-172-6D flight simulator is set in natural plains scenery, where participants conduct their flight experiments using the simulated flight platform. Each participant performs five segments: take-off, level flight, left turn, right turn, and landing (as depicted in Figure 5). The average aircraft flight altitude is 1000 feet, and the average flight speed is 70 knots [18].

For the left and right turns, pilots follow visual flight rules (VFR) without specific speed or altitude requirements, ensuring they maintain at least a 10 s interval between maneuvers to avoid interference. The detailed flight parameters are as follows:

Take-off phase: The aircraft ascends to 400 feet at a speed of 60 knots.

Climb phase: The aircraft continues to ascend to an altitude of 1000 feet.

Cruise phase: The pilot performs left and right turns.

Descent phase: The aircraft descends back to 400 feet.

Landing phase: The aircraft gradually decreases speed and altitude for a smooth landing.

Throughout the flight, the pilots’ EEG data were continuously recorded using an EEG cap, while changes in cockpit instrumentation and flight attitude were recorded using video equipment.

After completing the experiment, the pilots filled out the NASA-TLX subjective questionnaire based on their flight experiences during the cruising and turning phases. The subjective questionnaire assesses six dimensions of psychological workload. For each dimension, the pilots rated their experiences on a scale from 0 to 10. By calculating the weighted average across different dimensions, the level of psychological workload experienced by the pilots during different flight phases was determined.

### 2.4. Data Processing

The collected pilot EEG data were first preprocessed to ensure accurate analysis. The various functions and operating states of the brain can be determined based on human behavior and cognition, allowing corresponding EEG signals to be identified for analysis. EEG signals are commonly categorized into four main frequency bands: δ wave, θ wave, *α* wave, and *β* wave, as shown in Table 2 [19].

To filter the EEG signals and eliminate the impact of high and low-frequency noise, FIR (finite impulse response) filters were employed within the 0.1–40 Hz range. This preprocessing step is crucial for isolating the relevant EEG signals and improving the accuracy of subsequent analyses [20,21].

**Figure 6:**
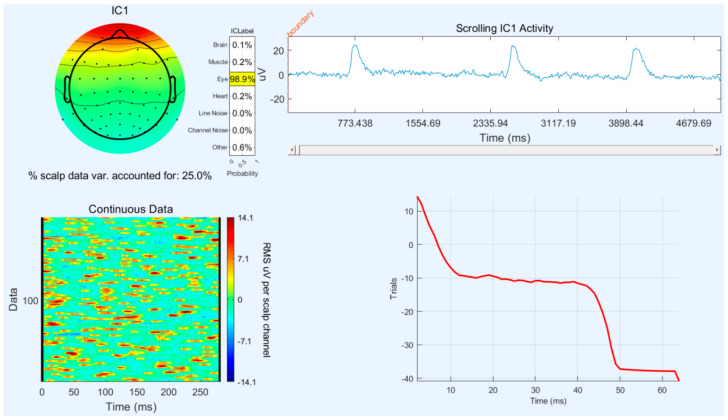
Artifact rejection—VEOG.

ICA is a powerful statistical technique used to decompose multichannel EEG signals into independent components. Each component represents a distinct source of neural activity contributing to the overall EEG signal [22]. During the ICA process, components associated with artifacts such as EMG (muscle activity) and EOG (eye movements) can be identified based on their characteristic patterns and frequencies. These components can then be effectively removed or filtered out from the EEG data. By isolating and removing artifacts through ICA, the quality and reliability of the EEG signals are significantly improved [23]. This allows more accurate analysis of brain activity patterns related to cognitive processes or specific tasks performed during the flight simulation. Figure 6 likely depicts the results of ICA, showing how independent components are extracted from the EEG data and how artifacts are separated from genuine brain signals. This visualization aids in validating the effectiveness of artifact removal and ensuring the integrity of the EEG data for subsequent analysis.

## 3. Results

### 3.1. Analysis of EEG Maps in the Left Turn Phase and Right Turn Phase

The EEG (electroencephalogram) data of the pilot were collected throughout the simulated aircraft operation process. During this simulation, the EEG signals specifically associated with beta waves were of particular interest during the pilot’s performance of left and right turns. These beta wave data underwent a series of preprocessing steps and characteristic extractions to isolate the relevant signals from the raw EEG data.

To enhance the quality of the EEG signals, particularly by reducing noise, a method called superimposed averaging was employed. This technique improves the signal-to-noise ratio, making the desired beta wave signals more distinguishable from background noise. After this enhancement, an EEG map was generated. This map provides a visual representation of the energy fluctuations in the beta wave signals across each electrode channel, allowing for a clear and comprehensive view of the pilot’s brain activity during the turning maneuvers [24].

**Figure 7:**
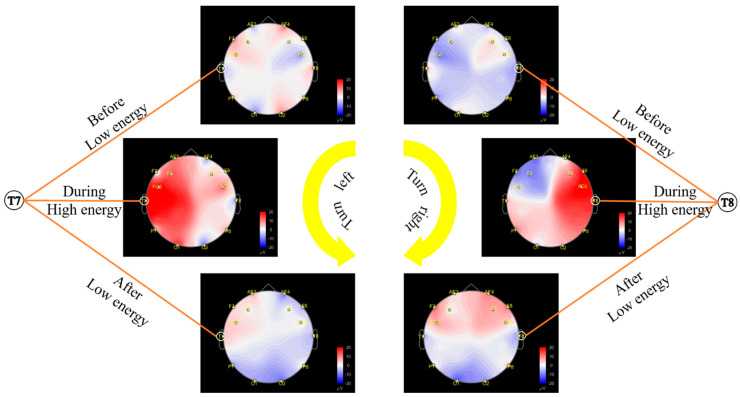
EEG map before, during and after turns.

During the cruise phase, the electrodes on the pilot’s EEG typically display light colors, indicating low signal energy levels. However, during a left turn, a notable surge in energy occurs at the T7 electrode, resulting in a darkening of its color, indicating increased neural activity levels. This surge is prominently displayed on the left side of the EEG map, showing heightened energy levels observed in the electrode channels. After the pilot completes the left turn maneuver, the energy levels at electrode T7 gradually diminish, resulting in a return to lighter coloration as the aircraft resumes level flight. Similarly, during right turns, there is an increase in energy at electrode T8, resulting in darker coloration at this electrode site. The right half of the EEG map shows elevated energy levels corresponding to this rise. Once the right turn is completed, the energy levels in each electrode gradually decrease again, indicating a return to level flight. These dynamic changes, including the shifts in energy levels and corresponding color changes, are illustrated in Figure 7, providing a clear visual representation of the pilot’s neural activity during different phases of the flight. However, the map does not facilitate quantitative analysis.

### 3.2. EEG Signal Correlation

During flight tasks, EEG signals exhibit variations across different electrodes. For correlation analysis, the study selected the beta waves of EEG signals recorded during the left and right turning phases. This study utilized Pearson correlation analysis to examine the EEG signals collected from the various electrodes [25,26]. The Pearson correlation coefficient formula used in this study is as follows:

(1) $$r=\frac{\sum_{i=1}^{n}\left(X_{i}-\bar{X})(Y_{i}-\bar{Y}\right)}{\sqrt{\sum_{i=1}^{n}{\left(X_{i}-\bar{X}\right)}^{2}}\sqrt{\sum_{i=1}^{n}{\left(Y_{i}-\bar{Y}\right)}^{2}}}$$

where $X_{i}$ and $Y_{i}$ represent the EEG signal values from two different electrodes at corresponding time points, while $\bar{X}$ and $\bar{Y}$ are the mean values of these signals over the selected time period. The resulting correlation coefficient r quantifies the linear relationship between the EEG signals from the two electrodes during the turning maneuvers. An r value close to 1 indicates a strong positive correlation, while an r value near −1 signifies a strong negative correlation. A value around 0 indicates no linear relationship between the signals of the electrodes.

**Figure 8:**
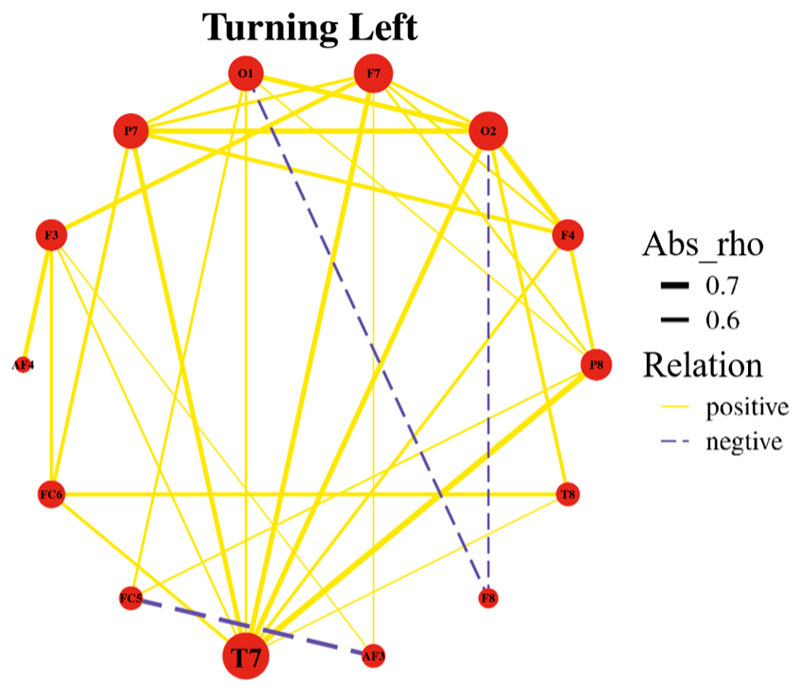
Spherical correlation graph for left turns.

In left-turn maneuvers, the highest degree of correlation was observed at electrode T7, indicating a strong correlation between the brain regions associated with T7 during left-turn maneuvers, as depicted in Figure 8.

**Figure 9:**
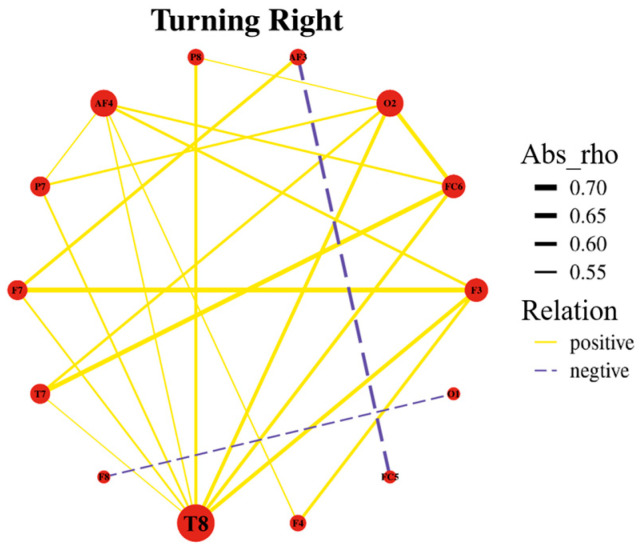
Spherical correlation graph for right turns.

In contrast, during right-turn maneuvers, the highest degree of correlation was observed at electrode T8, indicating strong correlations between the brain regions associated with T8 during these maneuvers. This finding highlights the necessity for further investigation into the relationship between turning directions and EEG signals, as depicted in Figure 9.

### 3.3. Extraction of EEG Energy Ratio

The study employed wavelet packet decomposition to extract four distinct rhythmic waves from the EEG signal. Within the frequency range of 0–30 Hz, the EEG signals were decomposed into delta waves (0–4 Hz), theta waves (4–8 Hz), alpha waves (8–12 Hz), and beta waves (12–30 Hz). This method decomposed the EEG signals, enabling the separation and analysis of these rhythmic waves. It yielded insights into varying states of consciousness during flight operations [27,28].

The mother wavelet selected for decomposition was db4, with each sub-band having a bandwidth of 2 Hz [29]. The formula is

(2) $$W_{j,k}=\langle x,\psi_{j,k}\rangle =\sum_{n}x\left[n\right]\psi_{j,k}\left[n\right]$$

where x represents the EEG signal. $\psi_{j,k}$ represents the wavelet basis function. $W_{j,k}$ represents the wavelet packet coefficients of the EEG signal at scale j and position k. $\langle x,\psi_{j,k}\rangle$ represents the inner product of the EEG signal and the wavelet basis function. Subsequently, the energy of the EEG signal within various frequency bands is computed utilizing the wavelet packet decomposition coefficients. The EEG energy is computed using the following equation:

(3) $$E_{j,k}={\sum_{j,k}\left|W_{j,k}\right|}^{2}$$

where $E_{j,k}$ represents the energy at the j scale and k position. Subsequently, the total energy for each rhythm is calculated by aggregating the energies of individual wavelets within specific frequency bands.

**Figure 10:**
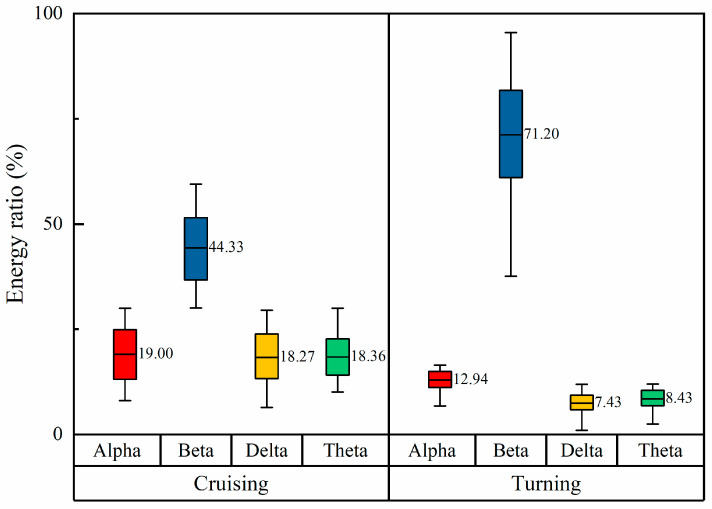
Energy ratios across different task phases.

For each type of rhythmic wave, the ratio of EEG energy to *β*/(*θ* + *α*) wave energy is computed to assess the proportion of EEG energy relative to the total energy. This method enables further analysis of how the distribution of EEG signal energy changes before and after task performance, providing deeper insights into variations across different task states. Figure 10 visually represents changes in EEG signal energy distribution across task phases.

In contrast to the cruising phase, during the turning phase, there is a significant increase in energy in the beta wave rhythm, with relatively lower energy levels observed in the delta, theta, and alpha wave rhythms, respectively.

**Figure 11:**
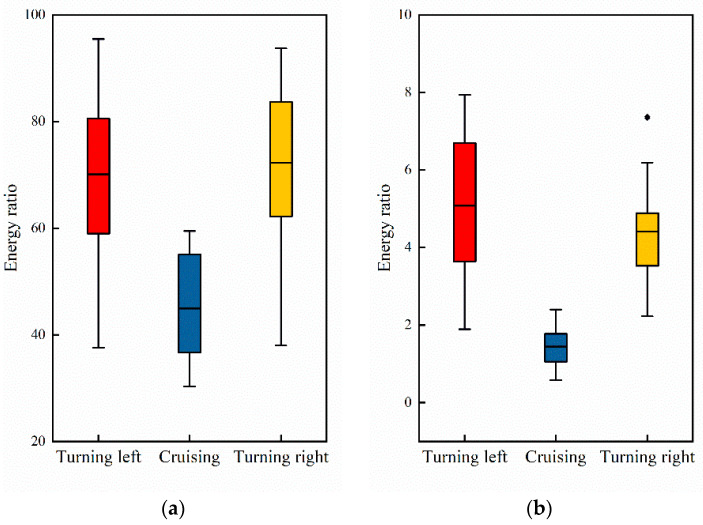
EEG energy characteristics. (**a**) Beta wave energy ratio; (**b**) *β*/(*θ* + *α*) wave energy.

Figure 11 illustrates the beta wave energy ratio and the *β*/(*θ* + *α*) wave energy of pilots’ EEG data during different flight phases. These metrics are higher during the turning phases and lower during the cruising phase. This indicates that pilots experience a greater cognitive load and increased brain activity during turns compared to cruising.

This finding is consistent with the understanding that turning maneuvers necessitate more intensive focus and decision-making, thereby increasing the psychological workload on pilots. During turns, pilots must constantly monitor and adjust the aircraft’s orientation, speed, and trajectory, which demands heightened attention and cognitive resources. Conversely, cruising typically involves more stable flight conditions, requiring less active control and allowing for reduced cognitive engagement.

Increased beta wave activity during turns reflects the brain’s response to heightened cognitive demands and stress, as beta waves are often associated with active thinking, focus, and problem-solving. Conversely, relatively lower beta activity during cruising suggests a more relaxed state, where the brain is engaged in routine monitoring rather than intensive problem-solving.

### 3.4. Extraction of EEG Entropy

EEG entropy serves as a metric for quantifying the complexity and randomness of EEG signals. It is commonly utilized in EEG signal analysis to assess the complexity of brain activity. In this study, we computed both Shannon entropy and sample entropy of the EEG signals to elucidate the variations in brain activity across different tasks [30,31,32].

**Figure 12:**
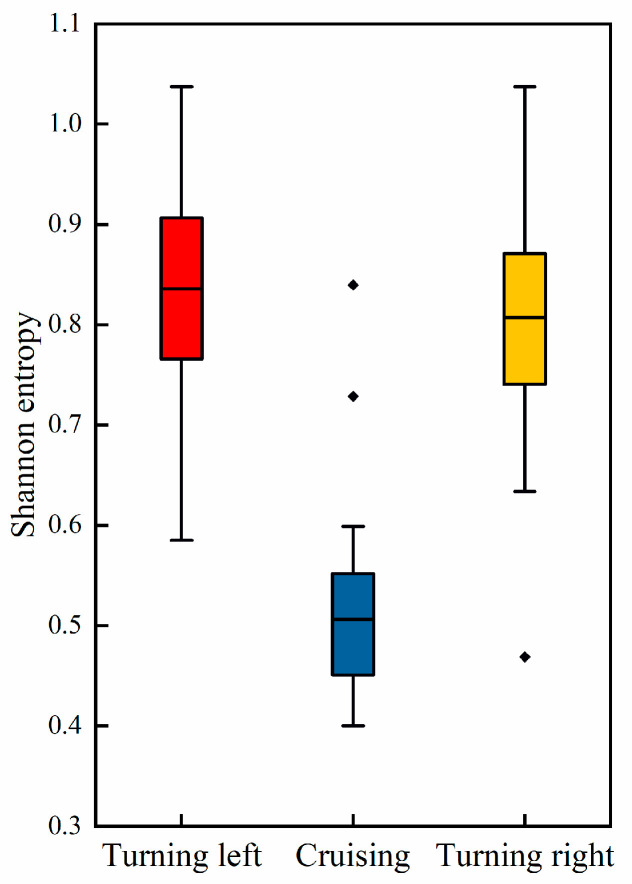
EEG Shannon entropy during different task phases.

Shannon entropy measures the average uncertainty or information content in the EEG signals, providing an indication of the overall complexity and unpredictability of brain activity. Higher Shannon entropy values suggest more complex and varied brain activity, while lower values indicate more predictable and less complex activity. The calculation formula is as follows:

(4) $$H(X)=-\sum_{i}p(x_{i})\log_{10}(p(x_{i}))$$

where H(X) represents the Shannon entropy of signal X, and $p(x_{i})$ represents the probability of the signal taking on value $x_{i}$. We computed the Shannon entropy associated with the pilot’s EEG signals in both cruising and turning phases, as depicted in Figure 12.

**Figure 13:**
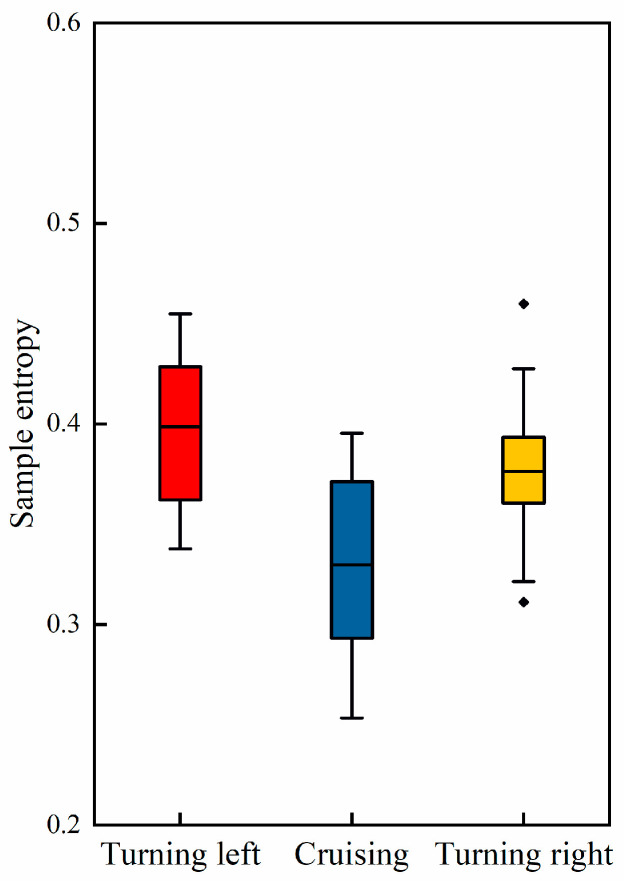
EEG sample entropy during different task phases.

Sample entropy is a nonlinear analysis method used to evaluate the complexity and regularity of time-series data. In EEG signal analysis, sample entropy is used to measure the repeatability or regularity of the signal at different time scales. Specifically, sample entropy can help identify patterns or events in EEG signals and assess the signal’s predictability and the system’s stability. The calculation of sample entropy typically involves defining a window size and a matching tolerance threshold to compare signal segments across different time scales. The calculation formula is

(5) $$SampEn(m,r,N)=-\ln\frac{A^{m}(r)}{B^{m}(r)}$$

where m is the embedding dimension, which defines the length of the sequences that are compared. r denotes the tolerance parameter. N is the length of the time series. $A^{m}(r)$ and $B^{m}(r)$ are counts of similar sequences in the time series: $A^{m}(r)$ is the count of sequences of length m+1 that are similar within tolerance r, and $B^{m}(r)$ is the count of sequences of length m that are similar within tolerance r, as depicted in Figure 13.

By separately computing these two types of entropy, the study aimed to obtain a comprehensive understanding of how brain activity complexity varies across different task phases. Compared to the cruising phase, pilots exhibit higher EEG entropy during turning phases. This increased entropy signifies a higher level of cognitive activity and complexity in brain function during turning maneuvers.

### 3.5. NASA-TLX Psychological Workload Assessment

Pilots’ psychological workload is subjectively assessed using the NASA Task Load Index (NASA-TLX). This tool collects pilots’ ratings of their psychological workload during different flight phases, specifically cruising and left and right turning phases. Pilots assess their workload across multiple dimensions, rating each dimension on a scale from “0” to “10”. A score of “0” indicates minimal workload, whereas a score of “10” indicates a high workload. This subjective assessment helps in understanding the perceived effort and stress experienced by pilots during different flight maneuvers and complements the objective EEG data analysis, providing a comprehensive view of pilots’ cognitive and psychological states [33].

**Figure 14:**
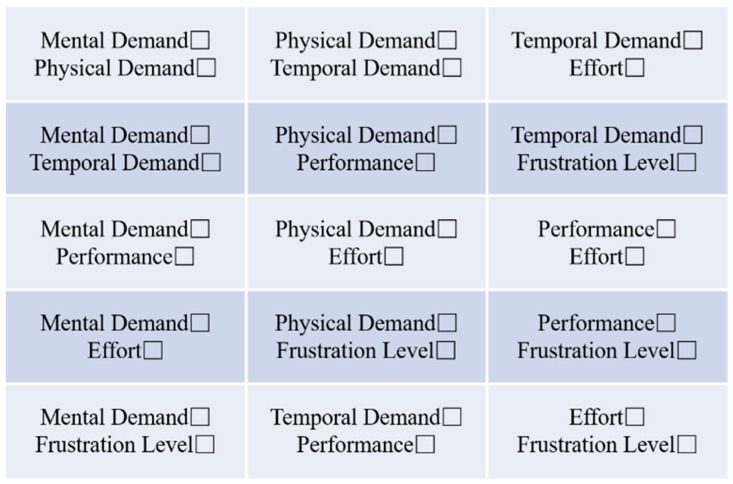
NASA-TLX weight test table.

Additionally, pilots must make pairwise comparisons across six dimensions, selecting the dimension they believe is more closely related to psychological workload, as shown in Figure 14. The formula for calculating the overall psychological workload is

(6) $$F=\sum_{i=1}^{6}M_{i}\times \frac{P_{i}}{15},$$

where F is the total score of the cognitive workload assessment. $M_{i}$ is the score of the subject in different dimensions. $P_{i}$ is the number of times different dimensions were selected in the weight testing table shown in Figure 14.

Subsequently, reliability and validity analyses were conducted on the weight proportions of different dimensions. The reliability (α=0.715) and validity (K=0.734) were found to be acceptable, ensuring the robustness of the weightings used in the workload assessment. The final psychological workload scores were 5.02 for the cruising phase and 6.13 for the turning phase. These results indicate that pilots experience a higher psychological workload during the turning phases than during the cruising phase, emphasizing the increased cognitive demands associated with maneuvering the aircraft during turns.

## 4. Psychological Workload Detection Model

### 4.1. Characteristic Parameter

A total of 2700 data sets, randomly selected from a pool of 3000 samples (comprising 1500 turn samples and 1500 cruising samples), were used for training. The remaining 300 data sets were reserved for testing. This approach ensures a balanced representation of both turning and cruising phases in the training data while reserving a separate set of data for evaluating the model’s performance.

Considering the variability in EEG data among different pilots, it was necessary to normalize the EEG energy ratio, Shannon entropy, and sample entropy to ensure consistency and comparability across individuals [34]. Normalization was performed using the following formula:

(7) $$X_{n}=\frac{X-X_{\min}}{X_{\max}-X_{\min}},$$

where X indicates the original characteristic parameter. $X_{n}$ is the normalized value of X. $X_{\min}$ is the minimum value of X in the dataset. $X_{\max}$ is the maximum value of X in the dataset.

Firstly, a normality test was conducted on the normalized characteristic parameters. The energy ratio of the EEG wave, *β*/(*θ* + *α*) wave energy, Shannon entropy, and sample entropy all satisfied the assumption of normal distribution (p>0.05) [35].

Subsequently, Pearson correlation analysis was used to calculate the correlation coefficients between Shannon entropy, sample entropy, and the energy ratio at various flight stages. This statistical method quantifies the degree of linear relationship between these variables, providing insights into how changes in entropy metrics correlate with variations in the energy ratio across different flight phases.

**Figure 15:**
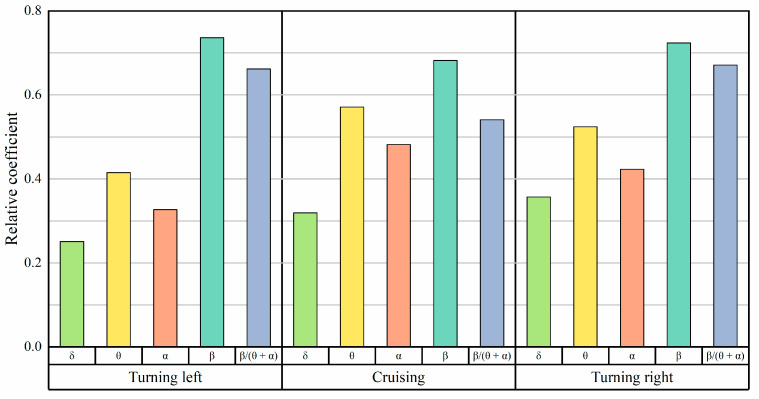
Pearson correlation coefficients between Shannon entropy and energy ratio at various flight stages.

**Figure 16:**
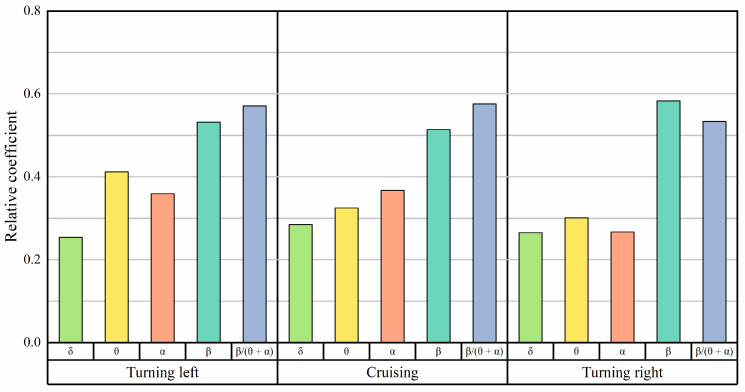
Pearson correlation coefficients between sample entropy and energy ratio at various flight stages.

Figure 15 and Figure 16 illustrate the correlation between Shannon entropy and sample entropy with the energy ratio of EEG. The beta band energy ratio and Shannon entropy exhibit the highest correlation among EEG characteristic quantities. The correlation coefficients are presented in Table 3.

**Figure 17:**
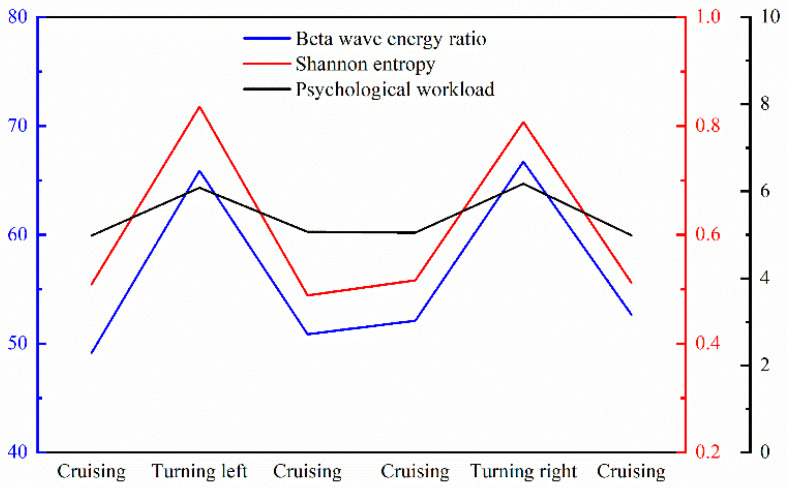
EEG characteristics and psychological workload during different flight maneuvers.

Figure 17 shows that, during the turning phases, the correlation between the beta wave energy ratio and the Shannon entropy of EEG signals is stronger than the correlation between the *β*/(*θ* + *α*) wave energy and the Shannon entropy of EEG signals. The Pearson correlation coefficient for left turns is 0.736, and for right turns, it is 0.724. During the cruising phase, the correlation coefficient between these two parameters is 0.682.

Figure 17 clearly demonstrates that both left and right turns are associated with a higher beta wave energy ratio, increased Shannon entropy, and greater psychological workload compared to the cruising phase. Furthermore, the trends of these parameters are consistent.

### 4.2. Detection Model

Our EEG characteristic detection model utilizes support vector machine (SVM). This method employs a radial basis kernel function to determine the flight phase in which the current EEG characteristics are located. This functionality guarantees high classification accuracy [36]. The radial basis kernel function $k\left(x_{i},y_{i}\right)$ is represented by the following function relationship:

(8) $$k(x_{i},y_{i})=x_{i}^{T}y_{i},$$

where $\left(x_{i},y_{i}\right)$ denotes the training samples. The following system of equations elaborates on the SVM process. The concept of a support vector machine involves finding the separating hyperplane that correctly divides the training dataset and has the maximum geometric margin. The separating hyperplane is

(9) λx+c=0,

where λ and c represent two parameters in separated hyperplanes. Subsequently, secondary optimization was conducted to determine the optimal boundary between the two variables. The calculation formula is as follows:

(10) $$\min_{\lambda ,c}\left[\frac{1}{2}{\left\|\lambda \right\|}^{2}\right],$$

Subsequently, the objective function is transformed into an unconstrained Lagrangian function.

(11) $$L(\lambda ,c,\omega )=\frac{1}{2}{\left\|\lambda \right\|}^{2}-\sum_{i=1}^{l}\omega_{i}\left[y_{i}\left(\left(\lambda \cdot x_{i}\right)+c\right)-1\right],$$

where $\omega_{i}$ represents the Lagrange multiplier. Based on the Karush–Kuhn–Tucker (KKT) conditions, we can derive the solution for the Lagrangian function.

(12) $$\left\{ \begin{array}{l} \frac{\partial L(\lambda ,c,\omega )}{\partial \lambda }=0\\ \frac{\partial L(\lambda ,c,\omega )}{\partial c}=0 \end{array}\right.,$$

The primal problem can be transformed into its dual form using Lagrange multipliers, resulting in

(13) $$\max L(\omega )=\sum_{i=1}^{l}\omega_{i}-\frac{1}{2}\sum_{i=1}^{l}\sum_{j=1}^{l}\omega_{i}\omega_{j}y_{i}y_{j}\left(x_{i}\cdot x_{j}\right),$$

where $\omega_{i}$ and $\omega_{j}$ represent the Lagrange multiplier. $x_{i}$ and $x_{j}$ are samples, and $y_{i}$ and $y_{j}$ are the corresponding labels.

The final discriminant function is as follows:

(14) $$f(x)=\mathrm{sgn}\left(\sum_{i=1}^{k}\omega_{i}y_{i}\left(x_{i}\cdot x\right)+c\right)$$

**Figure 18:**
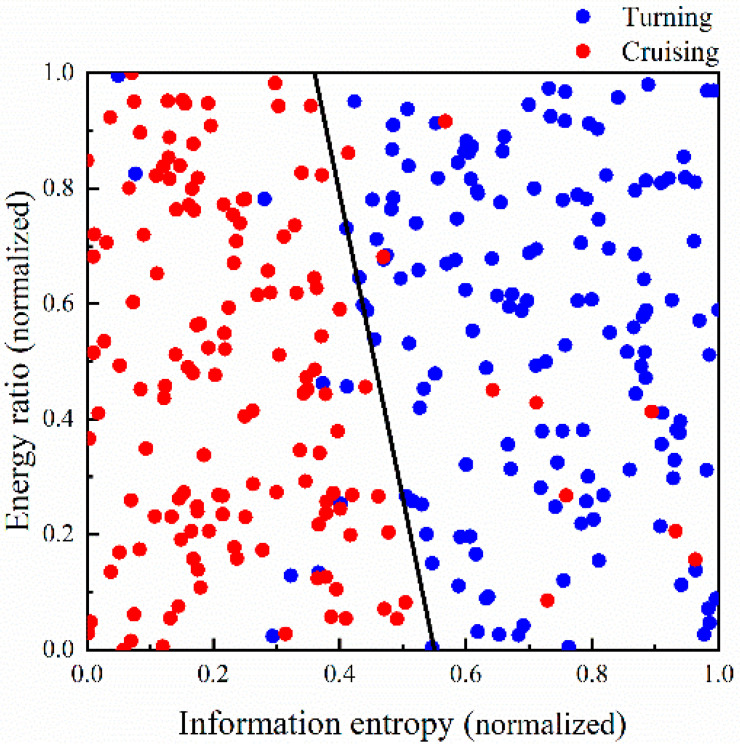
Classification results of psychological workload.

The NASA-TLX assessment indicates that pilots’ psychological workload is significantly higher during the turning phase compared to the cruising phase. The turning phase is characterized by high workload, whereas the cruising phase is characterized by low workload. The input EEG characteristics comprise the beta wave energy ratio and EEG Shannon entropy, which form the combination with the strongest correlation. The classification results of the pilots’ psychological workload in the test set are depicted in Figure 18.

The classification model results are depicted in Figure 18. The final classification accuracy on the training set is 98.92%. The classification accuracy on the test set is 93.67%.

## 5. Discussion

The study titled “Detection of Pilot’s Psychological Workload During Turning Phases Using EEG Characteristics” has clarified critical aspects of the cognitive workload experienced by pilots across various flight phases. The incorporation of electroencephalography (EEG) data for this purpose has created new opportunities to improve aviation safety and training methodologies. Several key points and implications stem from this research, warranting further discussion.

Interpretation of Results:

The observed increase in the ratio of beta wave energy and Shannon entropy during left and right turns indicates heightened cognitive activity. This aligns with existing literature that suggests beta waves are linked to active concentration and mental effort. The consistency of these findings across all pilots reinforces the robustness of EEG as a tool for assessing cognitive workload. The correlation analysis further supports these observations, demonstrating strong relationships between changes in EEG signals and specific flight maneuvers. This provides a quantitative basis for understanding the mental demands placed on pilots during various flight phases.

The subjective workload assessments using the NASA-TLX questionnaire aligned well with the EEG-based findings, thereby validating the objective measures. This dual approach strengthens the argument for using EEG in combination with traditional assessment tools to achieve a comprehensive understanding of pilot workload. The higher workload scores reported by the pilots during turns are consistent with the EEG data, thereby reinforcing the reliability of these measures in reflecting actual cognitive states.

The high classification accuracy of the SVM model in distinguishing between different workload states demonstrates the potential of machine learning in cognitive monitoring. The slight drop in accuracy from the training set to the test set is expected and underscores the need for further refinement and validation of the model with larger data sets. The SVM’s ability to classify workload states based on EEG data underscores the feasibility of developing real-time monitoring systems. Such systems could provide immediate feedback to pilots and support personnel, thereby enhancing decision-making processes during flights.

This in-depth analysis will not only enhance our understanding of the role of EEG in monitoring cognitive states but also offer a clearer perspective on the practical applications of these insights. Exploring how these systems can provide immediate feedback and support decision-making processes during flights may pave the way for significant advancements in aviation safety and training methodologies. Highlighting these practical applications may bridge the gap between theoretical research and its implementation, ultimately contributing to more effective and responsive aviation practices.

## 6. Conclusions

The study employed the EPOC + EEG headset to monitor signal variations in different brain regions of pilots during left and right turns. It analyzed EEG characteristics and the pilots’ psychological workload states using support vector machines for classification [37]. The study’s conclusions are summarized as follows:

**EEG Maps and Beta Wave Energy:** the EEG maps show a significant increase in beta wave energy at the T7 electrode during the left turn phase and at the T8 electrode during the right turn phase. During the cruising phase, the energy levels at various electrode points remain stable and relatively low.

**Entropy and EEG Complexity:** the results show that during aircraft turns, the entropy of EEG signals is higher compared to the cruising phase, indicating that EEG activity during the turning phase is characterized by increased complexity and irregularity.

**NASA-TLX and Psychological Workload:** statistical analysis using the NASA-TLX scale shows that pilots experience a higher psychological workload during turning phases compared to the cruising phase. This increased psychological workload may contribute to the complexity and irregularity of EEG signals during turning maneuvers.

**Support Vector Machine Classification:** By classifying EEG characteristics using a support vector machine, the psychological workload detection accuracy for the test set was 93.67%. These results indicate that this method has high accuracy in distinguishing the EEG characteristics associated with different levels of psychological workload, providing a reliable foundation for further application in the real-time monitoring of pilots’ psychological workload during flight operations.

### Future Work

The gender and age of pilots might influence the ratio of EEG energy and Shannon entropy. Additionally, pilots with varying levels of flying experience might experience different levels of psychological workload during cruising and turning phases. Therefore, further collection of EEG data from a diverse group of pilots is necessary to enhance the SVM dataset and improve classification accuracy. This will help gain a deeper understanding of pilots’ flying states and provide a theoretical basis for pilot training, thereby enhancing flight safety. Considering these additional factors will ensure a more comprehensive and accurate assessment of pilots’ cognitive and psychological states, leading to better training protocols and safer flight operations.

## Figures and Tables

**Table 1:** The corresponding brain regions for EEG electrodes.

Electrodes	Lobe
AF3, AF4, F3, F4 F7, F8, FC5, FC6	Frontal
T7, T8	Temporal
P7, P8	Parietal
O1, O2	Occipital

**Table 2:** The corresponding frequency and physiological meaning of EEG rhythms.

EEG Rhythm	Frequency (Hz)	Amplitude (μV)	Function	Main Location
*δ*	0.5~3	20~200	Sleep, hypoxia, etc.	Occipital and parietal areas.
*θ*	4~7	20~100	Burnout, sleep, etc.	Frontal and temporal areas.
*α*	8~13	10~100	Closed eyes, relaxation, etc.	Occipital lobe area.
*β*	14~30	5~20	Emotional tension, thinking activity, etc.	Temporal lobe and frontal lobe area.

**Table 3:** Pearson correlation coefficients.

EEG Entropy	Phases	δ Wave	θ Wave	*α* Wave	*β* Wave	*β*/(*θ* + *α*)
Shannon entropy	Turning left	0.251	0.415	0.327	0.736	0.662
	Cruising	0.319	0.571	0.482	0.682	0.541
	Turning right	0.357	0.524	0.423	0.724	0.671
Sample entropy	Turning left	0.254	0.412	0.359	0.532	0.571
	Cruising	0.285	0.325	0.367	0.514	0.576
	Turning right	0.265	0.301	0.267	0.583	0.534

## Data Availability

Given the ongoing research value of the data, scholars may reach out to the author to obtain access to it.
